# Adolescent elite skiers with and without cam morphology did change their hip joint range of motion with 2 years follow-up

**DOI:** 10.1007/s00167-018-5010-7

**Published:** 2018-06-07

**Authors:** Josefin Abrahamson, Anna Swärd Aminoff, Carl Todd, Cecilia Agnvall, Olof Thoreson, Pall Jónasson, Jón Karlsson, Adad Baranto

**Affiliations:** 1 Department of Orthopaedics, Institute of Clinical Sciences at Sahlgrenska Academy, University of Gothenburg, Gothenburg, Sweden; 2 Department of Occupational Orthopedics and Research, Sahlgrenska University Hospital/Mölndals Hospital, R-house, Floor 7, 431 80 Mölndal, Sweden; 3 Sportsmedicine Åre and Åre Ski High School, Åre, Sweden; 4 Orkuhúsið Orthopedic Clinic, Reykjavik, Iceland

**Keywords:** Femoroacetabular impingement, Athletes, Range of motion, Articular, Hip joint, Follow-up studies, Magnetic resonance imaging

## Abstract

**Purpose:**

To investigate how range of motion of the hips and the lumbar spine are affected by continued elite, alpine skiing in young subjects, with and without a magnetic resonance imaging verified cam morphology, in a 2-year follow-up study. The hypothesis is that skiers with cam morphology will show a decrease in hip joint range of motion as compared with skiers without cam, after a 2-year follow-up.

**Method:**

Thirty adolescent elite alpine skiers were examined at the baseline (mean age 17.3 ± 0.7 years) and after 2 years. All skiers were examined for the presence of cam morphology (*α*-angle > 55°) using magnetic resonance imaging at the baseline. Clinical examinations of range of motion in standing lumbar flexion and extension, supine hip flexion, internal rotation, FABER test and sitting internal rotation and external rotation were performed both at the baseline and after 2 years.

**Results:**

Skiers with and without cam morphology showed a significant decrease from baseline to follow-up in both hips for supine internal rotation (right: mean − 13.3° and − 10.9° [*P* < 0.001]; left: mean − 7.6° [*P* = 0.004] and − 7.9° [*P* = 0.02]), sitting internal rotation (right: mean − 9.6° and − 6.3° [*P* < 0.001]; left: mean − 7.6° [*P* = 0.02] and − 3.3° [*P* = 0.008]) and sitting external rotation (right: mean − 16.9° and − 11.4° and left: mean − 17.9° and − 14.5° [*P* < 0.001]) and were shown to have an increased left hip flexion (mean + 8.4° and + 4.6° [*P* = 0.004]). Skiers with cam were also shown to have an increased right hip flexion (mean + 6.4° [*P* = 0.037]). Differences were found between cam and no-cam skiers from baseline to follow-up in the sitting internal rotation in both hips (right: mean 3.25°, left: mean 4.27° [*P* < 0.001]), the right hip flexion (mean 6.02° [*P* = 0.045]) and lumbar flexion (mean − 1.21°, [*P* = 0.009]).

**Conclusion:**

Young, elite alpine skiers with cam morphology decreased their internal rotation in sitting position as compared with skiers without the cam morphology after 2 years of continued elite skiing.

**Level of evidence:**

II.

## Introduction

Hip and groin pains are common problems in the population, and especially in athletes. Femoroacetabular impingement syndrome (FAIS) has lately been given more attention as a major cause of hip pain [1, 2], and is defined as a clinical disorder in the hip joint where a combination of symptoms, clinical signs and imaging findings (abnormal morphology viewed on plain radiography, magnetic resonance imaging (MRI) or computed tomography) are manifested [3]. FAIS is either present as an abnormal morphology at the femoral head-neck junction (cam) or as an abnormality in the acetabular shape or orientation causing over-coverage of the femoral head (pincer) [4, 5]. These two can also be present together, as a mixed type. A result of this anatomic abnormal morphology, an impingement occurs when the femoral head-neck junction collides with acetabulum particularly during hip flexion and internal rotation (IR) [6]. In addition to pain, the scientific literature has shown growing evidence that a cam morphology might lead to decreased range of motion (ROM), damage to the cartilage, labrum tears and predispose to the development of hip joint osteoarthritis (OA) [4, 6–9]. Agricola et al. [7] reported that individuals with both an *α*-angle > 83° and limited hip joint IR (< 20°) were at high risk of end-stage OA within 5 years (adjusted odds ratio 25.21).

Previous studies have reported that patients with FAIS often have a motion and/or position related pain in the groin and/or hip joint, reduced hip flexion and IR and positive anterior impingement test /FADDIR test [3, 9, 10]. Agnvall et al. [11] found a significantly reduced hip flexion, supine IR and IR in three different sitting positions and a higher frequency of pain/discomfort in the FADDIR test, when comparing young adolescents with MRI-verified cam morphology and with no-cam [11].

The etiology of FAIS is not entirely known. However, it has been postulated that participating in high-impact sports during the growth period may predispose to cam morphological changes [12, 13]. It is suggested that vigorous sporting activity and repetitive micro-trauma to the proximal femoral physis [6] may lead to a reactive bone formation and the development of cam during growth spurt [14, 15].

Several studies have reported radiological changes of the cam morphology as high as 56–89% in young athletes participating in vigorous sporting activity, such as soccer [16, 17], basketball [18] and ice-hockey [19–21]. In healthy and less active asymptomatic populations, the prevalence of the cam morphology has been reported to be present in 10–50% on imaging [22, 23].

Alpine skiing is a forceful sport with great impact to the hip joints [24]. A recent study showed that young elite skiers are at a higher risk for having cam (49%) in comparison to young controls (19%) [25]. To our knowledge, it is unclear how the cam morphology affects the ROM in the lumbar spine and the hips over time with continuous vigorous sporting activity, e.g. alpine skiing.

The aim of the present study is to investigate how the ROM in the hip joints and the lumbar spine is affected by continued elite, alpine skiing in young subjects with and without an MRI-verified cam morphology after 2-year follow-up. The hypothesis is that young, elite alpine skiers with cam morphology will show a decrease in hip joint ROM as compared with young, elite alpine skiers without cam, after 2-year follow-up.

## Materials and methods

All students (*n* = 36, grade 1–2, 16–18 years) attending the Åre Ski Academy were invited to participate in this prospective, cohort study at baseline in 2014 and during follow-up in 2016. One subject was excluded at baseline due to FAIS-surgery. Thirty subjects (13 females and 17 males) were available for final analysis. Reasons for the reduced number of subjects were that only data from skiers who participated both at baseline and the follow-up were used and difficulties to get the skiers to be present at the investigations or failure to attend appointments despite several attempts. Participation was totally voluntary and informed written consent was given by all individuals. For participants younger than 18 years, informed written consent was also obtained from one parent.

The inclusion criteria were students at the Åre Ski Academy, training and competing at elite level. Participants were excluded if they were pregnant or had a history of previous surgery to the back, pelvis and/or hip.

The present study was approved by the Regional Ethical Review Board in Gothenburg at the Sahlgrenska Academy, Gothenburg University, Gothenburg, Sweden, (ID number: 692-13).

### MRI examination

At baseline, 2014, all participants underwent MRI examinations of both hips at the Radiological Department at Östersund Hospital, Sweden. No intra-articular contrast was used. The MRI was performed on a GE Optima 450 Wide 1.5T (Milwaukee, USA) using a coil surface HD 8ch Cardiac array by GE. Most cam morphological changes are in the anterosuperior head-neck junction [5, 6]. Therefore, the *α*-angle was measured at seven clockwise positions in 30° intervals, from 9 o’clock (posterior) to 3 o’clock (180°, anterior) to determine morphological findings at the femoral head-neck junction.

The *α*-angle was measured, according to Nötzli et al. between the femoral neck axis and a line from the center of the femoral head to a point where the contour of the femoral head-neck junction exceeds the radius of the femoral head [2]. The *α*-angle defines the presence of the cam morphology and previous studies have used a threshold of 55–60° [20, 21, 26]. In the present study, an *α*-angle of 55° or above is considered as a cam morphology.

The MRI scans were evaluated and measured unidentified by one experienced radiologist together with a resident radiologist according to a standardized protocol. The interobserver reliability (intraclass correlation, ICC) of the *α*-angle measurement was previously reported to be 0.75 [11].

### Clinical examination

The clinical examinations were carried out at Åre Ski Academy, Östersund, Sweden, following a standardized protocol, according to Agnvall et al. [11], both at baseline and at the follow-up. All clinical examinations were performed by two examiners (co-authors CA and ASA) in a specific order. Intra- and interobserver tests for all physical examination measurements have previously been reported to be good to excellent, ICC 0.77–0.78 and 0.83–0.94, respectively [11].

**Fig. 1:**
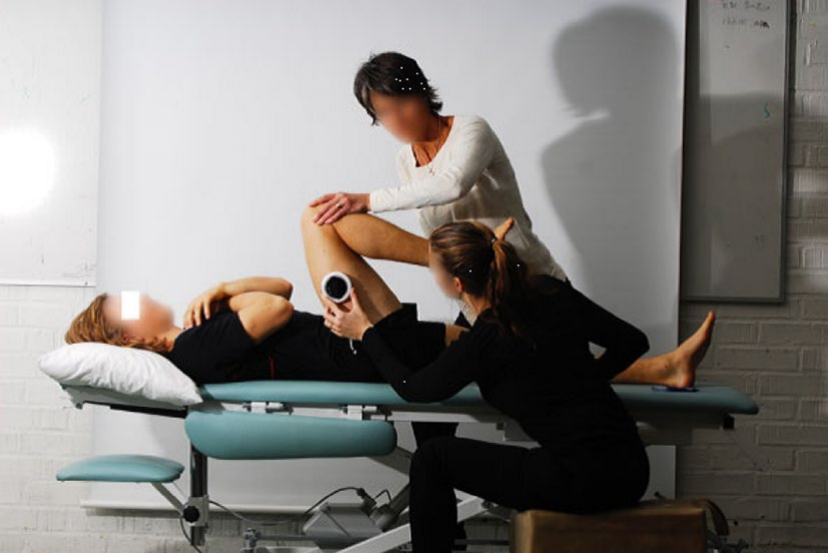
Passive flexion in the hip joint

**Fig. 2:**
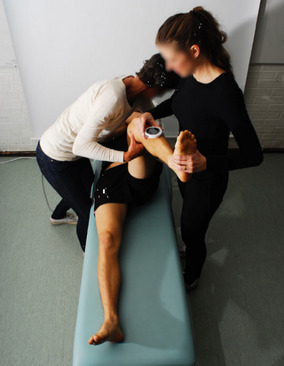
Passive supine internal rotation in the hip joint

**Fig. 3:**
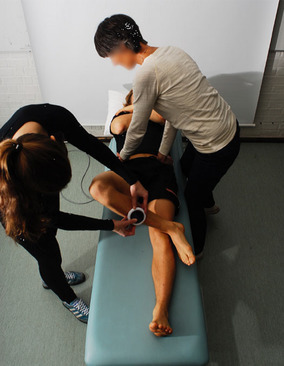
FABER test

**Fig. 4:**
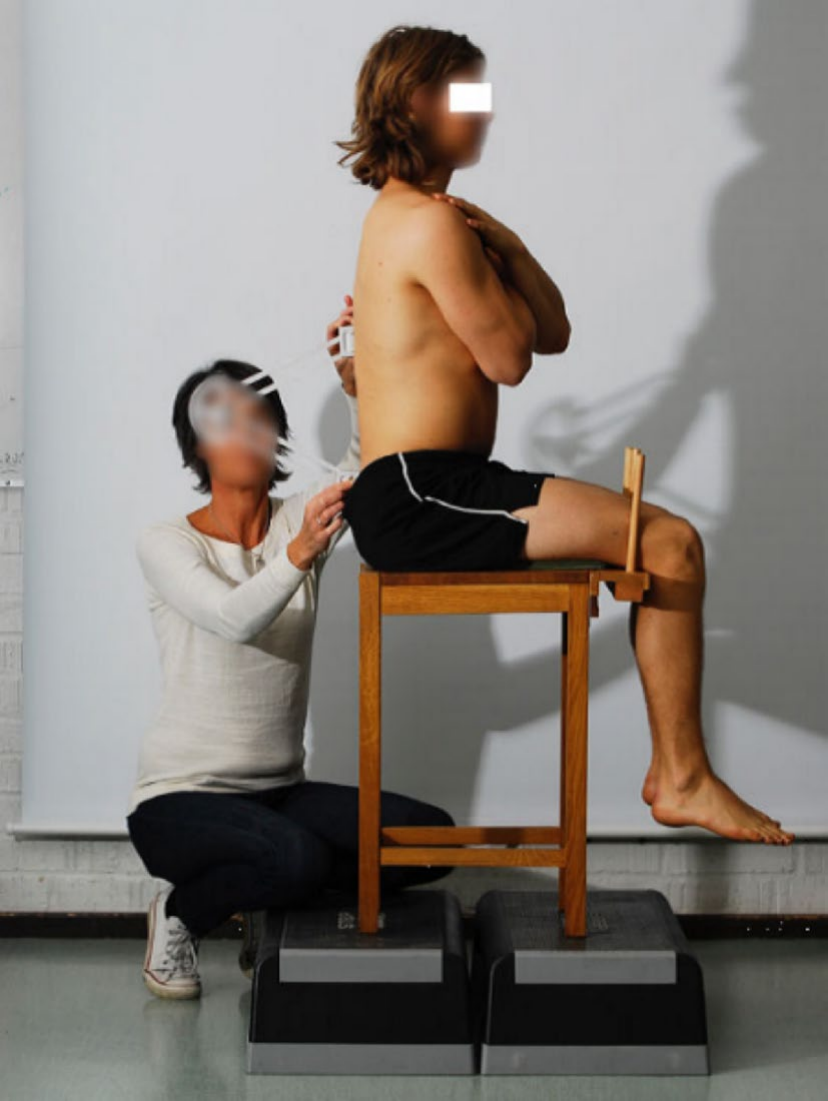
Neutral lumbal sitting position

All participants were first examined in a standing position with both feet together and arms hanging by their sides. A non-invasive measurement of lumbar flexion and extension was performed with a modified Debrunner Kyphometer (Protek AG, Bern, Schweiz) [27]. Secondly, the participants were examined in the supine position. Hip flexion (Fig. 1), hip internal rotation (IR) in 90° hip- and kneeflexion (Fig. 2) and FABER (flexion, abduction, external rotation) test (Fig. 3) were measured using a Digital Goniometer (DG) (HALO medical devices, Australia) [28] together with a Universal Goniometer (UG) with extended arms, 40 cm [29]. If the angles between the two devices differed, the angle measured by the UG was used for the final analysis. Final examinations were in a sitting, neutral position measuring passive hip IR and external rotation (ER) (Fig. 4) using a DG together with a UG as described previously. All measurements were recorded in degrees (°). We refer to the original work for a full description of the technical aspects [11].

### Statistical analysis

The data were analyzed using IBM SPSS Statistics for Windows, version 25.0 (Armonk, NY: IBM Corp). The description of continuous data was expressed in terms of the means and standard deviation (SD). The normal distribution of the data was tested with a Kolmogorov–Smirnov test. A paired *t* test was used to compare continuous data between baseline and the follow-up and an independent *t* test was used to evaluate the differences between the cam and no-cam group. The hips are analyzed separately, i.e. hips with an *α*-angle > 55° in the right hip (but not in the left hip) were included in the cam group when analyzing the results for the right hip, and similarly for the left hip. When analyzing the lumbar spine measurements, an *α*-angle > 55° in any hip were included in the cam group. Categorical data were expressed as frequencies and percentage and a Chi-square test was used for these data. All tests were two-sided, and the significance was set at *P* < 0.05.

## Results

A total of 56 hips in 30 (13 females and 17 males) adolescent elite skiers were available for the final analysis, of which 19 hips (34%, 9 right and 10 left) in 14 subjects (47%) had a cam morphology (*α*-angle > 55°) at baseline. Hips lost for final analysis were due to bad imaging quality and therefore inability to interpret the MRI scans. Baseline characteristics for the study population are summarized in Table 1. All follow-ups were at 24 months. The cam group consisted of four females (right: 3; left: 2) and 10 males (right: 6; left: 8), whilst the no-cam group consisted of nine females and seven males. The two groups were similar in terms of age, gender and BMI. All skiers continued to ski at elite level during follow-up time. The skiers lost to follow-up were similar regard to age, gender, height, weight, BMI, prevalence of hip deformities or symptoms.

**Table 1:** Baseline characteristics for all subjects stratified by group

	All subjects (*n* = 30)	Cam (*n* = 14)	No-cam (*n* = 16)	*P* value
Age, years	17.3 (0.7)	17.3 (0.8)	17.3 (0.6)	n.s.^a^
Female sex, *N* [%]	13 [43]	4 [27]	9 [60]	n.s.^b^
Height, cm	173 (8.0)	176 (8.5)	171 (7.1)	n.s.^a^
Weight, kg	68 (9.3)	69.7 (9.2)	65.7 (9.3)	n.s.^a^
Body mass index, kg/m^2^	22.5 (2.0)	22.6 (1.7)	22.3 (2.3)	n.s.^a^

Values are mean (SD) unless specified

^a^Independent sample *t* test

^b^Chi-square test

Tables 2, 3 and 4 summarize lumbar spine, right hip and left hip ROM differences between baseline and follow-up in both the cam and no-cam group. Both the cam and no-cam group had significant decrease in hip ROM for supine IR in both hips, sitting IR, sitting ER and an increase in the left hip flexion. The cam group had a significant increase for the right hip flexion which appeared different from the no-cam group. In contrast, the no-cam group was shown to have a decrease in the right FABER test, which was not found in the cam group.

**Table 2:** Differences in lumbar ROM in subjects with and without cam morphology, at baseline and follow-up

Outcome	Baseline	2 years	Change (Δ)	*P* value^a^
Lumbar flexion (*N* = 28)	34.1 (7.2)	33.2 (9.2)	0.9	n.s
Cam (*n* = 13)	34.5 (2.0)	32.9 (1.9)	1.6*	n.s
No-cam (*n* = 15)	33.8 (1.9)	33.4 (2.8)	0.4	n.s
Lumbar extension (*N* = 28)	− 57.6 (9.2)	− 60.7 (7.5)	3.0	n.s
Cam (*n* = 13)	− 58.3 (3.0)	− 62.3 (2.1)	4.0	n.s
No-cam (*n* = 15)	− 57.1 (2.1)	− 59.3 (1.9)	2.2	n.s

Values are presented in degrees as mean and (SD). *P* value indicates statistical significant difference between baseline and the 2 years follow-up (*P* < 0.05)

^a^Paired *t* test *indicates statistical difference with independent *t* test between cam and no-cam from baseline to 2 years follow-up (*P* < 0.05)

**Table 3:** Differences in the right hip ROM in subjects with and without cam morphology, at baseline and follow-up

Outcome right hip	Baseline	2 years	Change (Δ)	*P* value^a^
Supine hip flexion (*n* = 28 hips)	120.5 (8.2)	122.9 (8.2)	2.4	n.s
Cam (*n* = 9 hips)	118.2 (2.6)	124.7 (3.2)	6.4*	**0.037**
No-cam right (*n *= 19 hips)	121.6 (1.9)	122.1 (1.8)	0.4	n.s
Supine hip IR (*n* = 28 hips)	29.8 (8.1)	18.1 (7.7)	11.7	< **0.001**
Cam (*n* = 9 hips)	27.9 (2.2)	14.4 (2.5)	13.4	< **0.001**
No-cam (*n* = 19 hips)	30.7 (2.0)	19.8 (1.7)	10.9	< **0.001**
Supine FABER (*n* = 28 hips)	67.1 (6.5)	59.3 (7.9)	7.9	< **0.001**
Cam (*n* = 9 hips)	71 (2.0)	65.8 (2.8)	5.2	n.s
No-cam (*n* = 19 hips)	65.3 (1.4)	56.2 (1.3)	9.1	< **0.001**
Sitting hip IR (*n* = 28 hips)	35.8 (9.5)	28.4 (9.0)	7.4	< **0.001**
Cam (*n* = 9 hips)	33 (2.6)	23.4 (2.6)	9.6*	< **0.001**
No-cam (*n* = 19 hips)	37.1 (2.3)	30.8 (2.0)	6.3	< **0.001**
Sitting hip ER (*n* = 28 hips)	37.4 (5.9)	24.3 (5.6)	13.1	< **0.001**
Cam (*n* = 9 hips)	39.2 (2.8)	22.3 (1.9)	16.9	< **0.001**
No-cam (*n* = 19 hips)	36.5 (1.0)	25.2 (1.3)	11.4	< **0.001**

Values are presented in degrees as mean and (SD)

* IR* internal rotation,* ER* external rotation

Bold style indicating statistical significance difference between baseline and the 2 year follow-up (*P* < 0.05)

^a^Paired *t* test

*Indicates statistical difference with independent *t* test between cam and no-cam from baseline to 2 years follow-up (*P* < 0.05)

**Table 4:** Differences in the left hip ROM in subjects with and without cam morphology, at baseline and follow-up

Outcomes left hip	Baseline	2 years	Change (Δ)	*P* value^a^
Supine hip flexion (*n* = 28 hips)	120.8 (7.3)	126.7 (7.9)	5.9	< **0.001**
Cam (*n* = 10 hips)	119.3 (2.7)	127.7 (2.6)	8.4	**0.004**
No-cam (*n* = 18 hips)	121.6 (1.6)	126.1 (1.9)	4.6	**0.004**
Supine hip IR (*n* = 28 hips)	31.5 (9.9)	23.7 (11.4)	7.8	< **0.001**
Cam (*n* = 10 hips)	28.8 (2.7)	21.2 (3.8)	7.6	**0.004**
No-cam (*n* = 18 hips)	33 (2.5)	25.1 (2.6)	7.9	**0.02**
Supine FABER (*n* = 28 hips)	63.1 (10.3)	62.2 (8.8)	0.9	n.s
Cam (*n* = 10 hips)	64.6 (1.4)	65.2 (2.7)	0.6	n.s
No-cam (*n* = 18 hips)	62.2 (2.9)	60.5 (2.1)	1.7	n.s
Sitting hip IR (*n* = 28 hips)	37.4 (10.8)	32.6 (12.1)	4.9	< **0.001**
Cam (*n* = 10 hips)	34.3 (3.9)	26.7 (3.2)	7.6*	**0.02**
No-cam (*n* = 18 hips)	39.2 (2.5)	35.8 (2.9)	3.3	**0.008**
Sitting hip ER (*n* = 28 hips)	37.0 (5.7)	21.0 (3.6)	16.0	< **0.001**
Cam (*n* = 10 hips)	38.6 (2.1)	20.7 (0.8)	17.9	< **0.001**
No-cam (*n* = 18 hips)	36.2 (1.2)	21.2 (1.0)	14.5	< **0.001**

Values are presented in degrees as mean and (SD)

*IR* internal rotation,* ER* external rotation

Bold style indicating statistical significance difference between baseline and the 2 year follow-up (*P* < 0.05)

^a^ Paired *t* test

*Indicates statistical difference with independent *t* test between cam and no-cam from baseline to 2 years follow-up (*P* < 0.05)

There was a significant difference from baseline to follow-up between the cam and the no-cam group (Tables 2, 3, 4). The cam group had a greater decrease in lumbar flexion (mean − 1.21°, [*P* = 0.009]) and sitting IR in both hips (right: mean − 3.24°; left: mean − 4.27° [*P* < 0.001]) and a greater range in the right hip flexion (mean + 6.02° [*P* = 0.045]). No other significant differences were shown between the groups.

## Discussion

The most important findings in the present study were that adolescent skiers decreased their hip IR and ER regardless if they had MRI-verified cam morphological changes or not after 2 years follow-up. Young skiers with cam also increased their hip flexion and, additionally, were shown to have a statistical greater decrease in sitting IR and larger increase in the right hip flexion, from baseline to follow-up, as compared with the no-cam skiers. This was, in part, similar to the study by Agnvall et al. [11] where skiers with cam had significantly lesser IR in both supine and sitting positions as compared with the no-cam skiers. These minor differences between cam and no-cam, in the present study, might be explained by the late fusion of the pelvic bones. Partial fusion of the iliac crest occurs from the age of 15–22 years, with complete union in all individuals by the age of 23 years [30]. One may speculate that the acetabulum permits slight movement before fusion and therefore the cam morphological change does not affect the hip ROM. As the fusion progresses (i.e. the participants gets older) the acetabulum might not be as compromising and the hip ROM may therefore be affected.

The hypothesis in this present study was that skiers with MRI-verified cam morphology will show a decrease in hip joint ROM as compared with no-cam skiers after 2 years follow-up. This was true for hip IR in sitting position, but rejected for all other measurements that were shown to be similar between the groups or, as for the hip flexion, did increase instead of decrease. These results might be caused by early sporting participation with an adaption to the athletic activity (i.e. extra-articular hip conditions, e.g. soft tissue pathologies, and/or muscular stiffness) rather than as a response to a cam morphology.

Current literature highlights a lack of follow-up studies investigating hip ROM in populations with the cam morphology. Hip IR is the most common ROM measurement in studies of FAIS, as this suggests to be an important clinical finding in the presentation of FAIS [3]. Despite that supine IR did decrease from baseline (28–33°) to the follow-up (14–25°) in both groups, these results are consistent with earlier studies, in athletes (11–30°) [18, 31–33] and in individuals with radiological cam morphology (16–28°) [17, 34, 35].

The present study reported that the alpine skiers were shown to have reduced hip IR and ER from baseline, with a mean age of 17 years, to the follow-up, with a mean age of 19 years. This corresponds to the natural change of hip ROM that can be seen in both the normal population [36] and athletes [18, 33]. Siebenrock et al. [18] showed a decrease in hip IR when stratified into age groups (regardless cam or not), where 13–15-year-old basketball players had a mean IR of 23.4° as compared with 16–21-year-old players who had only 13.6°. They suggested a physiological loss of IR attributable to decreasing femoral neck anteversion during growth. Moreover, Manning and Hudson [33] compared junior soccer players (mean age 17.6 years) to senior soccer players (mean age 26.3 years) and found senior soccer players to be less flexible in hip flexion, IR and ER. In the study by Sankar et al. [36], who examined hip ROM in a normal population of 2–17-year-olds, found a trend toward lesser hip ROM with higher age in almost every participant. This was more pronounced in the male population. An interesting aspect is that these studies showed less flexibility also in flexion, as compared with the skiers in the present study that showed an increase in hip flexion. Though, the present result matches the review by Andersen and Montgomery [24], who reported alpine skiers had significantly better flexibility as compared to non-athletes in a hip flexion/extension testing. If the increased flexion is a response to the lesser hip IR and ER or to the highly dynamic ski performance and training that is performed in primarily a squatting position, is still unclear and needs further investigation.

Hip ER, in the present study, was shown to be in line with earlier studies in athletes [17, 32, 37] when compared at baseline, whilst the results from the follow-up are approximately 10° lower for both the cam and no-cam group. This may be due to an actual reduction in the hip joint ROM or muscular stiffness in the hip rotators, as a response to the alpine skiing [17].

The FABER test is commonly used as a diagnostic test in patients with hip and/or groin pain and is considered positive if the pain is reproduced and/or there is a decrease in ROM as compared with the non-affected leg [38]. The most common method to measure hip ROM with the FABER test is with a stick between the lateral femoral condyle on the test side and the examination table. However, this method has not been able to distinguish between cam and no-cam morphology [20]. In the present study, the hip ROM in FABER test was measured with a digital goniometer and the results were shown to conflict. The no-cam group had significantly lesser angle in the right hip from baseline to the follow-up, this was not shown for the cam group. In the left hip, none of the groups reached significant levels. Moreover, when comparing the groups from baseline to follow-up, there was no difference in either hip. Some previous studies, using this angle-measuring method, could not either report an association between hip ROM in the FABER test and participants with cam morphology [11, 32]. As the intra- and interobserver reliability was shown to be good to excellent in the present study [11], the likelihood of measurement errors due to the examiners is reduced. One may, therefore, speculate if the FABER test should be a predictor for hip ROM in patients with hip/groin pain, regardless of using the stick or the goniometer.

None of the groups did show any change from baseline to follow-up for the lumbar spine measurements. However, when comparing the groups from baseline to follow-up, a statistical difference was shown for LF. This may be due to a type-II error because of the relatively limited cohort, and the difference of 1.21° in the LF between the cam group and the no-cam group, for which the *P* value approaches significance, and similarly so for the sitting IR and right hip flexion. Furthermore, one must also take into consideration that these hip ROM differences between cam and no-cam were minor (3–6°) and did not reach the minimally detectable change (MDC) of 7° that Tak et al. [17] did calculate.

It appears that there is a discrepancy between studies investigating hip ROM and its association to cam [17, 19, 32, 34, 39]. This might rather be related to different measuring methods than actual ROM differences, and therefore, makes it difficult to compare results and draw final conclusions. The present study showed a significant difference after 2 years between the cam group and the no-cam group in sitting IR but not in supine IR. This may suggest that measuring hip IR in sitting position could be more sensitive to distinguish between cam and no-cam. Studies of hip IR in sitting position are sparse. Agnvall et al. [11] showed a significant difference for hip IR between cam and no-cam in young subjects in three different sitting positions. In contrast, Brunner et al. [39] failed to find any difference when comparing ice-hockey players with symptomatic FAIS, asymptomatic FAIS and no FAIS. The method in their study differs from the present study as they used an examination chair with a standard load of 5 kg applied on a lever arm, which passively moved both legs into hip IR. In this position, maximum IR was held for 30 s and then was measured bilaterally with an inclinometer. Moreover, they also included both types of FAIS: cam and pincer. The sitting position makes it possible to control and minimize counter-movements in the lumbar spine and pelvis, therefore, giving the results the possibility of greater reliability. Many patients with cam report difficulties and pain associated with prolonged sitting. Therefore, this position in combination with hip IR may have another impact on the hip as compared to that in the supine position. To better understand how hip ROM is affected by an abnormal morphology (e.g. cam), the activity that is related to their symptoms requires further research.

Several limitations of this study need to be acknowledged. Firstly, the skiers were only examined for cam morphology defined as an *α*-angle > 55° at the baseline. If, and how, hip morphology may or may not have been changed throughout the 2 years remains unclear. Secondly, the skiers were not examined for hip morphological findings (e.g. pincer, acetabular orientation) other than *α*-angle, which might affect the hip ROM. Audenaert et al. [40] proposed in their study the importance of examining the general hip morphological characteristics, especially femoral anteversion and acetabular coverage, and referred to the possibilities of an earlier collision between the femur and acetabulum in individuals with greater femoral retroversion. Thirdly, the present study only examined the hip ROM relative to cam, regardless of the presence of pain in the hip/groin or the lumbar spine. If the skiers had pain at baseline but not at the follow-up, or vice versa, the results might be affected false negative/positive. Furthermore, some studies have reported that patients with previous or present symptom/pain in the hip/groin are shown to have a lesser hip ROM as compared to asymptomatic persons [17, 34, 41]. It has previously been shown by Sadeghisiani et al. [42] in their systematic review that asymmetrical and limited hip IR and total hip rotation appear to be more common findings in patients who also present with low back pain.

The accuracy and interpretation of both radiological and clinical measurements is always dependent on the examiner, which is a limitation in itself. The present study attempted to limit such a variation by using only two radiologists and two collaborating examiners for the clinical examinations, alongside testing for intra- and inter-reliability.

The size of the sample group is a limitation, and a larger one might have revealed a greater difference between the cam and no-cam groups. However, the homogeneity of the present study may also be viewed as a strength, with respect to the sample of adolescent elite skiers is homogenous with respect to loading and levels of activity.

The clinical relevance of the present study highlights that elite alpine skiing during adolescence may cause hip ROM changes. These changes might be an adaption of soft tissues to the loads that alpine skiing requires, a physiological change during growth and/or an answer to the presence of a cam morphology in the hip joint.

## Conclusion

Adolescent elite alpine skiers changed their hip ROM after 2 years of continued skiing and the presence of MRI-verified cam morphology did correlate with a greater decrease in internal rotation in sitting position.

## Abbreviations

FAIS: Femoroacetabular impingement syndrome
MRI: Magnetic resonance imaging
ROM: Range of motion
IR: Internal hip rotation
ER: External hip rotation
LF: Lumbar flexion
LE: Lumbar extension
UG: Universal goniometer
DG: Digital goniometer
